# Digital Resilience of Clinical Nurses Across Hospital Tiers: A Multicenter Cross‐Sectional Study in China

**DOI:** 10.1155/jonm/4555487

**Published:** 2026-10-29

**Authors:** Ming Yu, Ronghui Geng, Rong Yu, Mengjia Zhou, Xiaoli Fan, Lingling Jiang

**Affiliations:** ^1^ Department of Nursing Affiliated Rudong Hospital of Xinglin College Nantong University Nantong 226001 Jiangsu China ntu.edu.cn

**Keywords:** AI literacy, clinical nurses, cross-sectional study, digital resilience

## Abstract

**Aim:**

To investigate the status of digital resilience among clinical nurses in China and to analyze its influencing factors, thereby providing a reference for promoting the application of artificial intelligence technology in healthcare.

**Design:**

A descriptive, cross‐sectional study.

**Methods:**

From August to September 2025, 680 clinical nurses were recruited from three hospitals in Nantong City, Jiangsu Province, China (one tertiary *A* hospital, *n = *220; two tertiary *B* hospitals, *n = *460). Data were collected using a general information questionnaire, the Artificial Intelligence Literacy Scale, the Perceived Organizational Support Scale, the General Self‐Efficacy Scale, and a clinically adapted version of the Digital Resilience Scale. Multiple linear regression (enter method) was performed to identify influencing factors.

**Results:**

The total score of digital resilience among clinical nurses was 123.68 ± 10.43 (out of 195). Nurses in the tertiary A hospital scored significantly higher than those in tertiary B hospitals (125.17 ± 6.49 vs. 123.05 ± 11.80, *t = *3.020, *p* = 0.003). Multiple linear regression revealed that perceived organizational support (*β = *0.621), hospital tier (*β = −*0.335), AI literacy (*β = *0.272), monthly income (*β = *0.126), monthly night shifts (*β = −*0.091), educational level (*β = *0.069), and self‐efficacy (*β = *0.066) were significant factors influencing digital resilience (all *p* < 0.05), collectively accounting for 76.3% of the total variance (adjusted *R* ^2^ *= *0.763).

**Conclusion:**

This multicenter study revealed significant disparities in digital resilience across hospital tiers. Tertiary A hospital nurses scored highest, followed by secondary hospital nurses, while tertiary B hospital nurses scored lowest. These differences were mainly driven by AI literacy, organizational support, and self‐efficacy. Hospital tier also moderated the relationship between organizational support and digital resilience, with stronger effects in lower‐tier hospitals.

**Implications for Nursing:**

Nursing administrators should implement tier‐specific strategies to strengthen digital resilience. Tertiary A hospitals need advanced AI training and leadership development. Secondary and tertiary B hospitals require foundational digital infrastructure and organizational support, including structured AI training, mentorship, and technical assistance. Differentiated resource allocation across hospital tiers will facilitate successful AI integration into clinical nursing practice. Future research should investigate the mechanisms underlying these tier differences and evaluate tier‐specific interventions.

## 1. Introduction

Artificial intelligence (AI) is fundamentally reshaping societies worldwide, transforming industries, economies, and the ways people live, work, and interact [1, 2]. In response to this profound shift, digital resilience has emerged as a critical individual capacity. Defined as the ability to adapt, learn, and continuously adjust in the face of digital disruptions, digital resilience equips individuals to navigate technological uncertainties, engage in proactive learning, and maintain psychological well‐being amid rapid digital change [3, 4]. Evidence indicates that individuals with higher digital resilience are better able to cope with the pressures of technological transitions and to enact adaptive behaviors that promote both personal and professional growth [5, 6].

In healthcare settings, AI applications are rapidly expanding across clinical scenarios. For instance, AI‐driven clinical decision support systems have been used to assist with medication dosing decisions and reduce medication errors [7, 8]; predictive analytics tools have been employed to identify deterioration trends in high‐risk patients [9, 10]; and tele‐nursing and wearable devices have enabled home‐based monitoring for patients with chronic conditions [11]. Additionally, AI has reduced nurses’ administrative burdens by automating tasks such as documentation and supply tracking [12].

As the largest group of healthcare professionals and frontline implementers of these technologies, nurses play a pivotal role in the successful adoption and integration of AI tools into daily clinical practice [13, 14]. Consequently, nurses’ digital resilience has become a critical factor influencing not only the effective implementation of AI technologies but also the quality and safety of patient care [15, 16]. Nurses with stronger digital resilience are more likely to embrace AI tools, adapt to workflow changes, and maintain high‐quality care delivery during digital transitions [17].

Despite its importance, systematic research on digital resilience among clinical nurses remains notably scarce. A small number of studies have assessed digital resilience among nursing students, with one study reporting that 83.99% of nursing students belonged to the “high digital resilience group” [18, 19]. These studies have identified several influencing factors among students, including gender, family income, professional identity, and peer support [18]. However, the student context differs fundamentally from clinical practice: nursing students learn in structured academic environments with dedicated digital skills training, while clinical nurses must acquire and apply digital competencies amidst heavy workloads, shift schedules, and direct patient care responsibilities [20]. Research has shown that while nursing students report high confidence in basic digital skills, they struggle to translate these competencies into clinical contexts [21]. The digital tools used in clinical practice, such as electronic health records, monitoring systems, and tele‐nursing platforms, differ markedly from those commonly found in educational settings. These contextual differences suggest that findings from student populations cannot be directly generalized to practicing nurses, underscoring the urgent need for empirical investigation of digital resilience specifically among clinical nurses.

To systematically investigate the mechanisms underlying clinical nurses’ digital resilience, this study draws upon Bandura’s social cognitive theory (SCT) (Figure 1), which posits that human behavior emerges from the reciprocal interaction of personal factors, behavioral patterns, and environmental influences. This theory has been widely applied in nursing research on digital technologies to explain how individuals adapt to new technological environments through observational learning, self‐efficacy, and outcome expectations [22–24]. Within this framework, AI literacy and self‐efficacy are conceptualized as personal cognitive resources that enable nurses to understand, evaluate, and effectively utilize digital technologies. Perceived organizational support represents the environmental dimension. It provides resources, recognition, and institutional policies [25]. These external conditions can either facilitate or hinder nurses’ adaptive responses to digital change [26]. Digital resilience itself is understood as the behavioral outcome of this dynamic interplay. We also draw on the conceptual model of digital resilience for clinical nurses proposed by Mo et al. [3], which, through Walker and Avant’s concept analysis methodology, systematically elucidated the core attributes of digital resilience (acquired behaviors, positive power, intrinsic force, and sustained dynamic feedback) and positioned individual, organizational, and technological factors as key antecedents. This integrated theoretical framework directly informs our study design: we position AI literacy and self‐efficacy as personal antecedents, perceived organizational support as an organizational antecedent, and digital resilience as the outcome, while also considering contextual factors such as demographic and professional characteristics.

**FIGURE 1:**
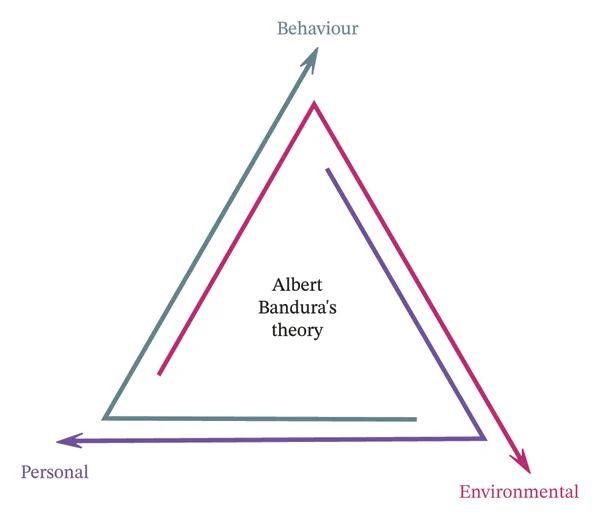
Bandura’s social cognitive theory (SCT).

In addition, hospitals in China are categorized into tiered levels based on resource availability, technological infrastructure, and clinical complexity. Tertiary A hospitals represent the highest tier with the most advanced digital systems and training resources, while tertiary B hospitals, particularly those in county‐level settings, often face greater resource constraints and less frequent exposure to cutting‐edge technologies [27]. Research indicates that hospitals at different tiers vary significantly in digital infrastructure, training opportunities, and technical support, and these systemic factors may shape nurses’ digital resilience in distinct ways [28]. However, empirical evidence on hospital‐level variations in digital resilience remains absent. Therefore, this study incorporates a comparative design to examine differences in digital resilience between nurses from tertiary A and tertiary B hospitals, thereby addressing the gap in understanding how practice environments influence this competency.

In summary, this study aims to: (1) describe the current state of digital resilience among clinical nurses in tertiary hospitals; (2) examine the associations between AI literacy, self‐efficacy, perceived organizational support, and digital resilience, based on the integrated theoretical framework; (3) compare digital resilience between tertiary A and tertiary B hospital nurses; and (4) identify demographic and professional factors associated with digital resilience. Based on the theoretical framework, we hypothesize that: (H1) AI literacy is positively associated with digital resilience; (H2) self‐efficacy is positively associated with digital resilience; and (H3) perceived organizational support is positively associated with digital resilience. The findings are intended to provide an empirical basis for developing targeted interventions to enhance nurses’ digital competencies across different hospital settings and to support the sustainable integration of intelligent nursing systems into healthcare delivery.

## 2. Methods

### 2.1. Participants and Settings

This was a descriptive, multicenter cross‐sectional study. From August to September 2025, clinical nurses were recruited from three hospitals in Nantong City, Jiangsu Province, China: one tertiary A hospital and two tertiary B hospitals. A total of 220 valid responses were collected from the tertiary A hospital and merged with 460 existing responses from the two tertiary B hospitals, resulting in a combined final sample of 680 participants.

Inclusion criteria were: (1) registered clinical nurses; (2) engaged in clinical nursing work for ≥ 6 months; (3) provided informed consent. Exclusion criteria were: (1) intern or trainee nurses; (2) nonclinical staff (e.g., nursing department administrators).

### 2.2. Sample Size

The sample size was determined a priori using *G Power* software (version 3.1.9.7) to accommodate both analytical objectives of this multicenter study [29]. For multiple linear regression (up to 12 predictors, *f* ^2^ *= *0.15, *α = *0.05, power = 0.95), the minimum required sample was 160, increased to 200 after accounting for attrition. For subgroup comparison (tertiary A vs. tertiary B hospitals, independent t‐test, Cohen’s *d = *0.5, *α = *0.05, power = 0.90), 172 participants per group were required, increased to 215 per group after attrition. Our tertiary B sample (*N = *460) already exceeded this requirement; we therefore collected 220 valid responses from a tertiary A hospital. The combined sample (*N = *680) exceeded all minimum requirements.

### 2.3. Demographic and Professional Information Questionnaire

A self‐developed Demographic and Professional Information Questionnaire was used to collect demographic and professional characteristics of the clinical nurses. The collected data included age, gender, educational level, years of work experience, professional title, monthly income, frequency of night shifts per month, and participation in AI‐related training.

### 2.4. Artificial Intelligence Literacy Scale (AILS)

The AILS, developed by Wang et al., was used to assess AI literacy among clinical nurses [30]. The scale consists of 12 items distributed across four dimensions: Awareness, Use, Evaluation, and Ethics, with each dimension containing three items. Responses are recorded on a 7‐point Likert scale ranging from 1 (strongly disagree) to 7 (strongly agree). The total score ranges from 12 to 84, with higher scores indicating greater AI literacy.

In the original development study, Wang et al. reported a Cronbach’s *α* of 0.87 for the total scale, indicating good internal consistency. In the present study, the Cronbach’s *α* for the total scale was 0.858, and the *α* values for the four subscales ranged from 0.82 to 0.89, indicating good to excellent internal consistency.

### 2.5. Nurse Perceived Organizational Support Scale

The Perceived Organizational Support Scale was originally developed by Chen Zhixia [31]. This scale has been widely used in nursing populations. It comprises two dimensions: emotional support (items 1–10) and instrumental support (items 11–13), totaling 13 items. Emotional support refers to the organization providing encouragement and emotional backing, while instrumental support refers to providing necessary tools and equipment. Items are rated on a 5‐point Likert scale (1 = strongly disagree to 5 = strongly agree). The total score ranges from 13 to 65, with higher scores indicating stronger perceived organizational support. The average item score determines the level: 1.00–2.33 (low), 2.34–3.66 (moderate), 3.67–5.00 (high).

In the original validation study, Zuo Hongmei et al. reported a Cronbach’s *α* of 0.905 for the scale. In the present study, the Cronbach’s *α* coefficient for the total scale was 0.980, indicating excellent internal consistency.

### 2.6. General Self‐Efficacy Scale (GSES)

The GSES is a unidimensional scale originally developed by Schwarzer and Jerusalem [19]. It contains 10 items rated on a 4‐point Likert scale (1 = not at all true to 4 = exactly true). The total score ranges from 10 to 40, with higher scores indicating higher levels of general self‐efficacy.

In the original Chinese validation study, Wang Caikang et al. reported a Cronbach’s *α* of 0.87 for the scale. In the present study, the Cronbach’s *α* coefficient for the total scale was 0.922, indicating excellent internal consistency.

### 2.7. Digital Resilience Assessment Scale

The Digital Resilience Scale was originally developed by Li Fanfan for nursing undergraduates [32]. In the original validation study with 442 nursing undergraduates, the scale demonstrated excellent internal consistency, with a total scale Cronbach’s *α* of 0.958, and subscale *α* values ranging from 0.900 to 0.944 (perceived digital risk: 0.944, cognitive coping strategies: 0.900, learning knowledge and skills: 0.944, overcoming digital stress: 0.901, adapting to the digital environment: 0.925).

Given the differences between academic and clinical settings, we adapted the scale for clinical nurses prior to data collection. Specifically, educational‐setting terminology was replaced with clinical‐setting equivalents (e.g., “school” was changed to “hospital,” “teacher” to “supervisor” or “head nurse,” “classroom” to “clinical setting,” and “fellow students” to “colleagues”). The adapted version was reviewed by three senior clinical nurses (with > 10 years of experience) to confirm the relevance and clarity of each item in the clinical context. The scale retained the original five dimensions and 39 items with Likert 5‐point scoring (1 = strongly disagree to 5 = strongly agree). Total scores range from 39 to 195, with higher scores indicating greater digital resilience.

In the present study, the Cronbach’s *α* for the total scale was 0.912, and the *α* values for the five subscales ranged from 0.82 to 0.94, indicating excellent internal consistency and supporting the scale’s reliability for group‐level comparisons among clinical nurses.

### 2.8. Data Collection

Data were collected via electronic questionnaires distributed through WeChat between August and September 2025 across three participating hospitals, including one tertiary A hospital and two tertiary B hospitals. A QR code linking to the questionnaire was disseminated to nursing staff in clinical departments at all three sites. The same data collection procedures were applied consistently across all hospitals to ensure comparability.

The preface of the questionnaire clearly outlined the study purpose, instructions for completion, and privacy protection measures. Participation was voluntary, and informed consent was obtained from all participants before they proceeded to complete the questionnaire. To ensure data completeness, the questionnaire incorporated a mandatory response feature, which prevented submission until all items were answered and provided real‐time reminders for any unanswered questions. An anonymous survey mechanism was implemented, and technical measures were applied to restrict each user to a single submission, thereby eliminating duplicate responses.

Invalid questionnaires, defined as those with a completion time of less than 120 s or showing patterned responses, were excluded. A total of 696 questionnaires were returned from the three hospitals. After exclusion of invalid responses (*n = *16), 460 valid responses from the two tertiary B hospitals and 220 valid responses from the tertiary A hospital were retained, yielding a combined final sample of 680 participants. The overall effective response rate was 97.70%.

### 2.9. Ethical Considerations

This study was approved by the Ethics Committee of Rudong Hospital (Approval No. 2022001). The study was conducted in accordance with the Declaration of Helsinki and its subsequent amendments or similar ethical standards. All participants signed an informed consent form and were informed of the study’s importance and goal by the researcher before the official investigation. Participants were told there would be no consequences if they chose to leave the research at any point. The researcher pledged to maintain the complete confidentiality and anonymity of the participants’ personal information.

### 2.10. Statistical Analysis

Data were analyzed using SPSS 26.0 (IBM Corp., Armonk, NY, USA). Descriptive statistics (frequencies, percentages, means, and standard deviations) were used to summarize demographic characteristics and scale scores. The Kolmogorov–Smirnov test was used to assess the normality of continuous variables, and all scale scores were found to be approximately normally distributed (*p* > 0.05), supporting the use of parametric tests.

For group comparisons, independent‐samples *t*‐tests were used for two‐group comparisons, and one‐way analysis of variance (ANOVA) was used for comparisons involving three or more groups. Categorical variables were compared using the *χ* ^2^ test. Pearson correlation analysis was employed to examine bivariate relationships among AI literacy, perceived organizational support, self‐efficacy, and digital resilience.

For multivariate analysis, multiple linear regression using the enter method was performed, with the total digital resilience score as the dependent variable [33]. All variables that demonstrated statistical significance in univariate analyses (*p* < 0.05) were simultaneously entered into the regression model as independent variables. Prior to running the regression, multicollinearity was assessed using variance inflation factor (VIF) and tolerance values, with VIF < 10 and tolerance > 0.10 considered acceptable. The coefficient of determination (*R* ^2^ and adjusted *R* ^2^ were reported to indicate the overall model fit.

A two‐tailed *p* < 0.05 was considered statistically significant for all analyses.

## 3. Results

### 3.1. General Information of Clinical Nurses

A total of 680 valid questionnaires were included in the final analysis. The demographic and professional characteristics of the participants are summarized in Table 1. The sample consisted predominantly of female nurses (98.7%), with the majority aged 31–40 years (65.1%). Over half held a junior college degree (56.6%) and a junior or below professional title (57.5%). Most nurses worked 4–9 night shifts per month (65.1%) and reported overtime work (95.4%). Notably, only 10.9% had participated in AI‐related training. Regarding hospital tier, 32.4% (*n* = 220) were from the tertiary A hospital and 67.6% (*n* = 460) from the two tertiary B hospitals.

**TABLE 1:** Demographic and professional characteristics of participants (*N* = 680).

Variable	Category	*n*	%
Gender	Male	9	1.3
	Female	671	98.7

Age (years)	≤ 25	136	20
	26–30	69	10.1
	31–40	443	65.1
	> 40	32	4.7

Education level	Secondary specialized school	199	29.3
	Junior college	385	56.6
	Bachelor’s degree or higher	96	14.1

Years of experience	≤ 3	201	29.6
	4–6	129	19
	7–10	116	17.1
	11–15	92	13.5
	≥ 15	142	20.9

Professional title	Junior or below	391	57.5
	Intermediate	271	39.9
	Senior	18	2.6

Monthly income (RMB)	≤ 4000	80	11.8
	4001–6000	269	39.6
	6001–8000	124	18.2
	≥ 8000	207	30.4

Monthly night shifts	0	136	20
	1–3	69	10.1
	4–9	443	65.1
	≥ 10	32	4.7

Overtime work (past 12 months)	Yes	649	95.4
	No	31	4.6

AI‐related training	Yes	74	10.9
	No	606	89.1

Hospital tier	Tertiary A	220	32.4
	Tertiary B	460	67.6

Abbreviation: AI = artificial intelligence.

### 3.2. Scores of Digital Resilience, Organizational Support, Self‐Efficacy, and AI Literacy

The mean scores for all key study variables are presented in Table 2. The total digital resilience score was 123.68 ± 10.43 (out of 195, scoring rate: 63.4%). Among the five dimensions, “overcoming digital stress” had the highest mean score (17.83 ± 2.27, item mean: 3.57), while “perceived digital risk” had the lowest (31.75 ± 2.94, item mean: 2.89), indicating that nurses demonstrated relatively strong stress‐coping abilities but were less capable of recognizing and identifying digital risks.

**TABLE 2:** Scores of digital resilience, organizational support, self‐efficacy, and AI literacy (*N* = 680).

Variable	*n* items	Min	Max	Total score	Item mean	Scoring rate (%)
Digital resilience (total)	39	99	151	123.68 ± 10.43	3.17	63.4
Perceived digital risk	11	26	42	31.75 ± 2.94	2.89	57.7
Cognitive coping strategies	6	16	24	19.41 ± 1.69	3.24	64.7
Learning knowledge and skills	10	26	40	31.52 ± 2.77	3.15	63
Overcoming digital stress	5	13	22	17.83 ± 2.27	3.57	71.3
Adapting to digital environment	7	18	28	23.57 ± 2.15	3.37	67.3
AI literacy (total)	12	36	65	50.23 ± 4.84	4.19	59.8
Awareness	3	9	17	13.04 ± 1.40	4.35	62.1
Using	3	9	16	12.11 ± 1.16	4.04	57.7
Evaluation	3	9	16	12.50 ± 1.20	4.17	59.5
Ethics	3	9	16	12.59 ± 1.19	4.2	60
Organizational support (total)	13	40	59	50.11 ± 4.29	3.85	77.1
Emotional support	10	30	44	37.28 ± 3.42	3.73	74.6
Instrumental support	3	10	15	12.84 ± 1.20	4.28	85.6
Self‐efficacy (total)	10	25	39	31.04 ± 1.55	3.1	77.6

*Note:* The scoring rate was calculated as (mean score/maximum possible score) × 100%. All items are positively scored.

Abbreviation: AI = artificial intelligence.

The mean AILS was 50.23 ± 4.84 (out of 84, scoring rate: 59.8%), with the highest score in the “awareness” dimension (13.04 ± 1.40) and the lowest in the “using” dimension (12.11 ± 1.16). The mean perceived organizational support score was 50.11 ± 4.29 (out of 65, scoring rate: 77.1%), with emotional support scoring higher than instrumental support (37.28 ± 3.42 vs. 12.84 ± 1.20). The mean self‐efficacy score was 31.04 ± 1.55 (out of 40, scoring rate: 77.6%).

### 3.3. Harman’s Single‐Factor Test

The first unrotated factor explained 26.803% of the total variance, well below the recommended threshold of 40%. This suggests that common method bias did not substantially threaten the validity of our findings.

### 3.4. Comparison of Digital Resilience Between Tertiary A and Tertiary B Hospitals

To examine hospital‐level differences in digital resilience, independent‐samples *t*‐tests were conducted comparing nurses from the tertiary A hospital (*n = *220) and tertiary B hospitals (*n = *460). As shown in Table 3, nurses in the tertiary A hospital demonstrated significantly higher total digital resilience scores than those in the tertiary B hospitals (125.17 ± 6.49 vs. 123.05 ± 11.80, *t = *3.020, *p* = 0.003).

**TABLE 3:** Comparison of digital resilience between tertiary A and tertiary B hospitals.

Dimension	Tertiary A (*n* = 220)	Tertiary B (*n* = 460)	*t*	p
Perceived digital risk	30.53 ± 1.92	32.34 ± 3.16	*−*9.215	**< 0.001**
Cognitive coping strategies	18.92 ± 1.16	19.66 ± 1.84	*−*6.359	**< 0.001**
Learning knowledge and skills	30.75 ± 1.57	31.90 ± 3.12	*−*6.402	**< 0.001**
Overcoming digital stress	20.27 ± 1.20	16.67 ± 1.65	32.16	**< 0.001**
Adapting to digital environment	24.70 ± 1.25	23.00 ± 2.27	12.583	**< 0.001**
Digital resilience (total)	125.17 ± 6.49	123.05 ± 11.80	3.02	**0.003**

*Note:* Bold values indicate statistical significance (*p* < 0.05). Italic values (*t*) denote test statistics.

At the dimension level, significant differences were observed across all five dimensions. Notably, tertiary A nurses scored significantly higher in “overcoming digital stress” (20.27 ± 1.20 vs. 16.67 ± 1.65*, t = *32.160, *p* < 0.001) and “adapting to the digital environment” (24.70 ± 1.25 vs. 23.00 ± 2.27, *t = *12.583, *p* < 0.001). However, tertiary A nurses scored significantly lower in “perceived digital risk” (30.53 ± 1.92 vs. 32.34 ± 3.16, *t = −*9.215, *p* < 0.001), “cognitive coping strategies” (18.92 ± 1.16 vs. 19.66 ± 1.84, *t = −*6.359, *p* < 0.001), and “learning knowledge and skills” (30.75 ± 1.57 vs. 31.90 ± 3.12*, t = −*6.402, *p* < 0.001). These findings suggest that while tertiary A nurses demonstrate higher overall digital resilience and stronger stress‐coping abilities, they perceive fewer digital risks and rely less on formal coping strategies, potentially reflecting greater digital proficiency and environmental familiarity.

### 3.5. Univariate Analysis of Factors Associated With Digital Resilience

Univariate analyses were performed to identify demographic and professional factors associated with digital resilience. As shown in Table 4, significant differences in digital resilience scores were observed across multiple variables, including hospital tier *(t = *3.020, *p* = 0.003), age (*F = *17.392, *p* < 0.001), educational level (*F = *160.595, *p* < 0.001), years of experience (*F = *27.929, *p* < 0.001), professional title (*F = *10.152, *p* < 0.001), monthly income (*F = *70.326, *p* < 0.001), monthly night shifts (*F = *33.414, *p* < 0.001), overtime work (*t = −*4.016, *p* < 0.001), and AI‐related training (*t = *6.687, *p* < 0.001). No significant difference was found based on gender (*p* = 0.686).

**TABLE 4:** Univariate analysis of factors associated with digital resilience (*N* = 680).

Variable	Category	*n*	Digital resilience score	*t/F*	*p*
Gender	Male	9	122.33 ± 5.83	*−0.405*	*0.686*
	Female	671	123.75 ± 10.48		

Hospital tier	Tertiary A	220	125.17 ± 6.49	*3.02*	**0.003**
	Tertiary B	460	123.05 ± 11.80		

Age (years)	≤** **25	136	120.47 ± 9.54	*17.392*	**< 0.001**
	26–30	69	122.17 ± 9.87		
	31–40	443	124.79 ± 10.18		
	>** **40	32	129.09 ± 11.43		

Education level	Secondary specialized school	199	119.10 ± 8.84	*160.595*	**< 0.001**
	Junior college	385	121.38 ± 9.01		
	Bachelor’s degree or higher	96	139.85 ± 8.59		

Years of experience	≤** **3	201	120.63 ± 9.92	*27.929*	**< 0.001**
	4–6	129	121.42 ± 8.95		
	7–10	116	121.33 ± 10.52		
	11–15	92	127.78 ± 9.69		
	≥** **15	142	129.25 ± 10.33		

Professional title	Junior or below	391	120.48 ± 8.24	*10.152*	**< 0.001**
	Intermediate	271	127.36 ± 10.92		
	Senior	18	135.67 ± 8.96		

Monthly income (RMB)	≤** **4000	80	117.53 ± 8.02	*70.326*	**< 0.001**
	4001–6000	269	121.56 ± 9.28		
	6001–8000	124	123.53 ± 8.84		
	≥** **8000	207	128.07 ± 10.58		

Monthly night shifts	0	136	129.96 ± 10.49	*33.414*	***< *0.001**
	1–3	69	124.58 ± 8.37		
	4–9	443	122.01 ± 9.96		
	≥ 10	32	121.25 ± 9.86		

Overtime work	Yes	649	123.39 ± 10.18	*−4.016*	**< 0.001**
	No	31	131.00 ± 12.82		

AI‐related training	Yes	74	131.15 ± 10.74	*6.687*	***< *0.001**
	No	606	122.83 ± 10.03		

*Note:* Bold values indicate statistical significance (*p* < 0.05). Italic values (*t*) denote test statistics.

Notably, nurses with higher educational levels (bachelor’s degree or higher: 139.85 ± 8.59) demonstrated substantially higher digital resilience than those with lower educational attainment (junior college: 121.38 ± 9.01; secondary specialized school: 119.10 ± 8.84). Similarly, nurses with higher monthly income (≥ 8000 RMB: 128.07 ± 10.58) scored significantly higher than those with lower income (≤ 4000 RMB: 117.53 ± 8.02). Nurses who had participated in AI‐related training also reported significantly higher digital resilience than those who had not (131.15 ± 10.74 vs. 122.83 ± 10.03, *p* < 0.001).

Pearson correlation analysis revealed that digital resilience was positively correlated with AI literacy (*r = *0.610, *p* < 0.001), perceived organizational support (*r = *0.747, *p* < 0.001), and self‐efficacy (*r = *0.143, *p* < 0.001).

### 3.6. Multiple Linear Regression Analysis of Factors Influencing Digital Resilience

To identify independent predictors of digital resilience, multiple linear regression using the enter method was performed. All variables that demonstrated statistical significance in univariate analyses (*p* < 0.05) were simultaneously entered as independent variables, with hospital tier coded as 1 = tertiary *A*, 2 = tertiary *B*.

The regression model was significant (*F = *183.139, *p* < 0.001) and explained 76.3% of the variance in digital resilience (adjusted *R* ^2^ *= *0.763). As shown in Table 5, multicollinearity diagnostics indicated no significant collinearity among predictors, with VIF values ranging from 1.021 to 3.154 (well below the threshold of 10).

**TABLE 5:** Multiple linear regression analysis of factors influencing digital resilience (*N* = 680).

Variable	*B*	*β*	*t*	*p*	VIF
(Constant)	13.124	—	2.37	*0.018*	—
Age	−0.403	−0.035	−1.103	*0.271*	2.816
Hospital tier (1 = Tertiary A, 2 = Tertiary B)	−7.457	−0.335	−17.012	**< 0.001**	1.11
Educational level	1.12	0.069	2.672	**0.008**	1.906
Years of experience	−0.172	−0.025	−0.751	*0.453*	3.154
Professional title	−0.862	−0.041	−1.38	*0.168*	2.513
Monthly income	1.269	0.126	5.013	**< 0.001**	1.798
Monthly night shifts	−1.101	−0.091	−3.791	**< 0.001**	1.651
Overtime work	0.275	0.005	0.284	*0.776*	1.072
AI‐related training	−1.115	−0.033	−1.539	*0.124*	1.343
AI literacy	0.62	0.272	10.913	**< 0.001**	1.782
Perceived organizational support	1.629	0.621	26.995	**< 0.001**	1.518
Self‐efficacy	0.448	0.066	3.479	**< 0.001**	1.021

*Note:* *R* ^2^ = 0.767, Adjusted *R* ^2^ = 0.763, *F* = 183.139, *p* < 0.001. Bold values indicate statistical significance (*p* < 0.05). Italic values (*t*) denote test statistics.

Perceived organizational support emerged as the strongest predictor (*β = *0.621, *p* < 0.001), followed by hospital tier (*β = −*0.335, *p* < 0.001; negative coefficient indicates tertiary A > tertiary B), AI literacy (*β = *0.272, *p* < 0.001), monthly income (*β = *0.126, *p* < 0.001), monthly night shifts (*β = −*0.091, *p* < 0.001), educational level (*β = *0.069, *p* = 0.008), and self‐efficacy (*β = *0.066, *p* < 0.001). Age, years of experience, professional title, overtime work, and AI training participation were not significant predictors in the final model.

## 4. Discussion

### 4.1. Status of Digital Resilience Among Clinical Nurses

The findings indicate that digital resilience among Chinese clinical nurses is at a moderate to moderately high level. This result is consistent with previous research on nursing students, although the scores in our clinical sample were comparatively lower [19]. Among the five dimensions, the highest scoring rate was observed in the “overcoming digital stress” dimension. The lowest was in the “perceived digital risk” dimension. This pattern suggests that nurses demonstrate relatively strong psychological resilience in managing pressures associated with digital work environments. However, their awareness of and preparedness for potential digital risks may represent a significant vulnerability in their overall digital competence.

Several factors may explain the relatively low performance in the “perceived digital risk” dimension. Current digital training in nursing tends to emphasize operational procedures and technical proficiency, such as electronic health record documentation and device operation. Systematic education on associated risks is often insufficient. These risks include data security, patient privacy protection, information ethics, and the risks of over‐reliance on technology [34]. In addition, the fast‐paced nature of clinical environments requires nurses to prioritize diagnostic and treatment tasks. This may limit their capacity to remain vigilant regarding latent safety threats arising from system flaws or improper use, such as alarm fatigue or misjudgments caused by information overload [35]. Another important observation is that nurses from tertiary A hospitals reported significantly lower perceived digital risk than those from tertiary B hospitals.

The “learning knowledge and skills” dimension scored at a moderate level. This reflects the ongoing challenge of keeping pace with rapid technological evolution. Digital knowledge acquired during formal nursing education may quickly become outdated. Upon entering clinical practice, nurses face heavy workloads. They often lack access to systematic, forward‐looking continuing education in digital competencies. This hinders their ability to update their skills [36]. Notably, only a small proportion of participants had participated in AI‐related training. This finding further underscores the educational gap. Compared with nursing undergraduates, the lower digital resilience observed in our clinical sample may reflect fundamental differences between academic and clinical settings. These differences include variations in learning environments, time availability, and access to structured digital education [37].

### 4.2. Factors Associated With Digital Resilience

#### 4.2.1. Perceived Organizational Support as the Strongest Predictor

Perceived organizational support emerged as the strongest predictor of digital resilience. This finding is consistent with our hypothesis. It also aligns with previous research demonstrating that organizational resources are fundamental to individual adaptive capacity [38]. The result supports SCT, which emphasizes that environmental factors play a critical role in shaping behavioral outcomes. Nurses who perceive strong organizational backing are better equipped with the psychological and material resources needed to navigate digital transitions. From a resource conservation perspective, organizational support may buffer against resource depletion during digital transformation [39]. This enables nurses to invest energy in proactive learning and adaptation rather than merely coping with change‐related stress.

### 4.3. Hospital Tier as a Significant Contextual Factor

Hospital tier emerged as a significant predictor of digital resilience. Nurses in tertiary A hospitals scored higher than those in tertiary B hospitals. This finding aligns with the resource‐based view of resilience. Higher‐tier hospitals typically have greater financial and technological resources. They also serve more diverse patient populations and offer more frequent exposure to advanced digital systems. These advantages may contribute to enhanced digital competencies and adaptive capacity [28].

However, the direction of this association warrants careful interpretation. Tertiary A nurses scored higher on overall digital resilience and on the “overcoming digital stress” dimension. Yet they scored lower on the “perceived digital risk” dimension. This paradoxical pattern may reflect a “competence‐confidence” effect. More digitally proficient nurses are more familiar with digital systems. As a result, they may perceive fewer risks. Less experienced nurses in tertiary B settings may perceive digital environments as more threatening. They may therefore actively seek more coping strategies. This interpretation is consistent with research on technology self‐efficacy. Higher competence reduces perceived threat and the need for external support [40].

### 4.4. AI Literacy as a Key Individual Factor

AI literacy demonstrated a significant positive association with digital resilience. This finding supports our hypothesis and is consistent with prior research on technology acceptance and digital competence [41].

Nurses with higher AI literacy can move beyond operational tasks. They can understand the underlying principles and data logic of intelligent systems [42]. This deeper understanding helps them troubleshoot more effectively when system failures occur. It also enables them to adapt when conflicts arise between human judgment and AI recommendations [43].

### 4.5. Self‐Efficacy as a Personal Cognitive Resource in Digital Adaptation

Self‐efficacy demonstrated a significant positive association with digital resilience, supporting our hypothesis and aligning with prior research in nursing populations. Among 823 nursing interns from eight tertiary hospitals in China, self‐efficacy was positively correlated with digital resilience (*r* = 0.590, *p* < 0.01) and partially mediated the relationship between digital literacy and digital resilience, accounting for 19.10% of the total effect [19]. This finding is consistent with Bandura’s SCT, which posits that individuals’ beliefs in their capabilities are stronger predictors of behavior and outcomes than their actual abilities [44]. In the digital health context, self‐efficacy has been identified as a critical psychological resource that enables nurses to confidently navigate digital tools, manage technical challenges, and adapt to evolving healthcare technologies [45]. Furthermore, nurses’ digital resilience is closely tied to self‐efficacy and learning capacity, as confidence in handling digital tasks and an ongoing commitment to update digital knowledge are prerequisites for resilient responses to technological change [46].

### 4.6. Demographic and Occupational Factors

Monthly income and educational level were positively associated with digital resilience. In contrast, monthly night shifts showed a negative association. These findings are consistent with resource‐based theories [47]. Higher income and education provide greater access to personal and professional development resources [48]. Frequent night shifts deplete cognitive and emotional resources, which are essential for adaptation.

## 5. Implications for Nursing Management and Practice

These findings have several practical implications.

Healthcare administrators should prioritize strengthening organizational support systems. This includes both instrumental and emotional support. Instrumental support refers to adequate digital infrastructure and technical assistance. Emotional support involves recognition of nurses’ efforts and psychological safety during transitional periods. Strong organizational support can serve as a foundational strategy for enhancing nurses’ digital resilience.

Nursing educators should develop structured AI literacy curricula. These curricula should emphasize deep understanding rather than superficial exposure. Incorporating case‐based and scenario‐based learning can help nurses build practical competence. This approach may better prepare them for real‐world digital challenges.

Given the hospital‐level disparities observed in this study, tier‐specific intervention strategies are needed. Lower‐tier hospitals may require additional investment in digital infrastructure and training resources. Such investments could help bridge the resilience gap between hospital tiers.

Human resource management should consider optimizing shift schedules. Reducing unnecessary night shift burden may help preserve nurses’ cognitive and emotional resources. These resources are essential for effective digital adaptation.

Fostering self‐efficacy is equally important. Progressive mastery experiences can build confidence. Peer support systems can provide encouragement and shared learning. Process‐focused feedback can guide improvement. These strategies may enhance nurses’ confidence in navigating digital challenges.

### 5.1. Limitations

Several limitations of this study should be acknowledged. The Digital Resilience Scale used in this study was originally developed for nursing undergraduates and, although adapted for clinical nurses, its factor structure has not been formally validated in this population; thus, the absolute estimates of digital resilience levels should be interpreted with caution. The cross‐sectional design precludes causal inferences, and the convenience sampling from three hospitals in a single city limits generalizability. Although Harman’s single‐factor test suggested that common method bias was not a serious concern, the possibility cannot be entirely ruled out given that all variables were self‐reported. Future research should develop a clinical nurse‐specific digital resilience scale and employ longitudinal, multicenter designs to validate our findings.

## 6. Conclusion

This multicenter cross‐sectional study found that digital resilience among clinical nurses in China was at a moderate level. Multiple factors significantly influenced digital resilience, with perceived organizational support emerging as the strongest predictor, followed by hospital tier, AI literacy, monthly income, monthly night shifts, educational level, and self‐efficacy. Notably, nurses in tertiary A hospitals demonstrated higher digital resilience than those in tertiary B hospitals. These findings suggest that healthcare administrators should prioritize strengthening organizational support, enhancing AI literacy through structured training, optimizing shift schedules to reduce night shift burden, and implementing tier‐specific interventions to address hospital‐level disparities.

## Author Contributions

Ming Yu: conceptualization, methodology, formal Analysis, investigation, writing–original draft, and project administration.

Xiaoli Fan: methodology, software, validation, investigation, data curation, and writing–review and editing.

Rong Yu: validation, investigation, and resources.

Mengjia Zhou: validation, investigation, resources, and visualization.

Ronghui Geng: investigation and resources.

Lingling Jiang: investigation and resources.

Lingling Jiang: conceptualization, resources, writing–review and editing, supervision, and funding acquisition.

## Funding

This work received no funding.

## Ethics Statement

This study was approved by the Ethics Committee of Rudong Hospital (Approval No. [2024] Ethics Review Approval (80), granted on October 28, 2024, valid until October 27, 2025). Data collection was conducted between August and September 2025, which fell entirely within the valid period of the ethics approval.

## Consent

The authors have nothing to report.

## Conflicts of Interest

The authors declare no conflicts of interest.

## Data Availability

The datasets generated and analyzed during this study are available from the corresponding author upon reasonable request.
